# Serum Levels of Fibroblast Growth Factor 21 Are Positively Associated with Aortic Stiffness in Patients with Type 2 Diabetes Mellitus

**DOI:** 10.3390/ijerph18073434

**Published:** 2021-03-26

**Authors:** Sin-Yi Huang, Du-An Wu, Jen-Pi Tsai, Bang-Gee Hsu

**Affiliations:** 1 Department of General Practice, Far Eastern Memorial Hospital, New Taipei City 22016, Taiwan; michellest7@gmail.com; 2 School of Medicine, Tzu-Chi University, Hualien 970374, Taiwan; despdu@yahoo.com.tw; 3 Division of Metabolism and Endocrinology, Hualien Tzu Chi Hospital, Buddhist Tzu Chi Medical Foundation, Hualien 970473, Taiwan; 4 Division of Nephrology, Department of Internal Medicine, Dalin Tzu Chi Hospital, Buddhist Tzu Chi Medical Foundation, Chiayi 62247, Taiwan; 5 Division of Nephrology, Hualien Tzu Chi Hospital, Buddhist Tzu Chi Medical Foundation, Hualien 970473, Taiwan

**Keywords:** aortic stiffness, fibroblast growth factor 21, carotid–femoral pulse wave velocity, type 2 diabetes mellitus

## Abstract

Aortic stiffness (AS), assessed using carotid–femoral pulse wave velocity (cfPWV), is associated with cardiovascular disease in type 2 diabetes mellitus (T2DM). The relationship between serum fibroblast growth factor 21 (FGF-21) and AS in T2DM patients was evaluated. Fasting serum FGF-21 levels of 130 T2DM patients were measured using an enzyme immunoassay kit. A validated tonometry system was used to measure cfPWV (>10 m/s indicated AS). Of these T2DM patients, 34.6% were defined as the AS group. T2DM patients with AS were older; exhibited higher systolic blood pressure, diastolic blood pressure, and body fat mass; higher triglyceride, fasting glucose, glycosylated hemoglobin, and creatinine levels; higher urine albumin-to-creatinine ratios and serum FGF-21 levels; and lower estimated glomerular filtration rates. The FGF-21 level (odds ratio = 1.005, 95% confidence interval: 1.002–1.009, *p* = 0.002) as well as systolic blood pressure was an independent predictor of AS and positively correlated to cfPWV values (β = 0.369, *p* < 0.001) in T2DM patients. For T2DM patients, serum FGF-21 level could be a predictor for AS.

## 1. Introduction

Inadequate control of glucose levels, dyslipidemia, inflammation, and endothelial dysfunction clinically presenting as aortic stiffness (AS) in diabetes mellitus (DM) patients have been reported to be risk factors for future atherosclerotic and cardiovascular (CV) events [1,2]. Among risk factors in DM patients, AS has been reported to result from vascular damage, remodeling, and dysregulation of endothelial collagen; inflammation can transmit extreme pulsatile energy into the microcirculation, thereby causing microvascular damage and ultimately resulting in target organ injuries, such as an increase in afterload of the left ventricle [2,3] and new-onset or long-term CV events, including coronary artery disease, stroke, peripheral arterial disease, and CV death [1,4]. The severity of AS is assessed by measuring carotid–femoral pulse wave velocity (cfPWV), which is the gold standard for evaluating arterial wall function and structure [5]. The results of a meta-analysis that involved the general population as well as patients with hypertension, end-stage renal disease, and DM showed that an increase in cfPWV values correlated with an increase in the pooled relative risks of total CV events, CV mortality, and all-cause mortality [6].

Circulating fibroblast growth factor 21 (FGF-21), being produced mainly by the liver and in relatively low amounts by adipose tissues, pancreatic β-cells, and skeletal muscles, was reported to exhibit insulin-sensitizing effects and to act as a modulator of glucose and lipid homeostasis [7]. FGF-21 has been demonstrated to physiologically modulate gluconeogenesis and lipolysis in the liver and adipose tissues during fasting and starvation after binding to its receptor and forming a complex with β-klotho; furthermore, studies have shown that administration of FGF-21 reduced body weight, dyslipidemia, and insulin sensitivity [8,9]. However, a growing body of evidence indicates a positive relationship between an increase in the serum levels of FGF-21 and obesity, dyslipidemia, insulin resistance, and DM [8]. Moreover, high serum levels of FGF-21 in DM patients were shown to be correlated with subclinical atherosclerosis [10] and a relatively high risk of CV events in short- and long-term studies [11,12,13].

Because evidence has shown the importance of cfPWV as a surrogate to predict future CV events, the application of a non-invasive, easy to learn approach, which could be performed in an outpatient setting to measure cfPWV by using applanation tonometry seemed necessary. A community-dwelling adult revealed that cfPWV was independently associated with CV mortality, coronary heart disease, and stroke at each quartile of increasing velocity [14]. Another study of DM patient’s follow-up for about 10 years showed that each increment of 1 m/s PWV independently increased about 1.8 times higher risk of all-cause and CV mortality [15]. Likewise, DM patients without known CVD were followed-up for about 9 years showed serum biomarkers indicative of vascular inflammation were associated with subclinical atherosclerosis (AS) and adverse CV events [1]. Of our previous studies, we found that cystatin C as well as adipocyte fatty acid binding protein correlated with AS in T2DM patients [16,17]. Therefore, to examine clinical valuable biomarkers associated with the diagnosis of AS could help with earlier diagnosis and treatment of subclinical CVD or even CV morbidity and mortality. Moreover, recent studies had shown that FGF-21 could be a potential predictor for fatty liver, dilated cardiomyopathy, insulin resistance, and cachexia [18,19,20,21]. However, the relationship between FGF-21 and AS in T2DM patients remained unclear, so we conducted this cross-sectional study to investigate the association between cfPWV and FGF-21 and its possible role as a predictor for AS in T2DM patients.

## 2. Materials and Methods

### 2.1. Patients

A total of 130 patients diagnosed with T2DM from November 2014 through March 2015 at a medical center in eastern Taiwan were enrolled. A standardized method of measuring blood pressure (BP) of the patients, who were seated for at least 10 min before the systolic BP (SBP) and diastolic BP (DBP) were recorded in the morning, was used. Patients were excluded if they had acute infectious diseases, acute cardiac diseases, active malignancy, or if they refused to provide informed consent. The study was approved by the Research Ethics Committee, Hualien Tzu Chi Hospital, Buddhist Tzu Chi Medical Foundation (IRB103-136-B). Informed written consent was obtained from all the patients.

### 2.2. Anthropometric Analysis

Body weights (BW), as well as heights (BH) of all the patients, were measured by the same operator when they were wearing light clothing and no shoes. Body mass index (BMI) was calculated as (BW)/(BH)^2^. Body fat mass was examined by the bioimpedance method with a 50 kHz single-frequency apparatus (Biodynamic-450; Biodynamics Corporation, Seattle, WA, USA) [16,17,22].

### 2.3. Biochemical Analyses

Fasting blood samples of approximately 5 mL were collected from the patients and immediately sent for biochemical examination by an autoanalyzer (Siemens Advia 1800; Siemens Healthcare GmbH, Henkestr, Germany). Random spot urine samples were sent for measurement of the ratio of albumin to creatinine (UACR) [16,22]. Values of FGF-21 were measured by enzyme immunoassay kits (Phoenix Pharmaceuticals, Inc. Burlingame, CA, USA) [22]. Renal function was manifested as estimated glomerular filtration rate (eGFR) calculated from the Chronic Kidney Disease Epidemiology Collaboration equation [23].

### 2.4. Measurements of cfPWV

We applied the pressure applanation tonometry (SphygmoCor system, AtCor Medical, New South Wales, Australia) to measure cfPWV [17,23]. The time interval between the R waves of the carotid and femoral arteries is simultaneously referred to with 10 continuous cycles of an electrocardiogram. The distance was obtained by subtracting the distance from the carotid measurement site to the sternal notch and from the sternal notch to the femoral measurement site. Then, the values of cfPWV were calculated by dividing the distance by the mean time between the two recorded points. A cfPWV > 10 m/s defined the aortic stiffness (AS) group and ≤ 10 m/s defined the control group, according to previous studies [16,17,24].

### 2.5. Statistical Analysis

The Kolmogorov–Smirnov test was used to examine the normality of continuous variables. Then these variables were expressed as means ± standard deviation or medians with interquartile ranges (IQRs) according to whether there is a normal distribution. Continuous variables were compared between the patients with AS and the control group, and the differences were analyzed using Student’s *t*-test (two-tailed) or Mann–Whitney *U* test according to the Kolmogorov-Smirnov test. Categorical variables were expressed as percentages and analyzed using the chi-square test. Predictors of AS in these patients were analyzed using multivariate stepwise logistic regression analysis by variables including age, body fat mass, SBP, DBP, TG level, fasting glucose level, HbA1c level, creatinine level, eGFR, UACR, and FGF-21 level. The factors that did not exhibit a normal distribution (TG level, fasting glucose level, HbA1c level, BUN level, creatinine level, UACR, and FGF-21 level) were logarithmically (log) transformed to achieve normality for subsequent linear regression analysis. The correlations between cfPWV and these variables were analyzed by using simple and multivariate stepwise linear regression analysis. A receiver operating characteristic (ROC) curve was applied to calculate the area under the curve (AUC) and to examine the optimal cut-off value of FGF-21 to predict AS in T2DM patients. Data were analyzed using SPSS for Windows (version 19.0; SPSS Inc., Chicago, IL, USA). A value of *p* < 0.05 indicated statistical significance.

## 3. Results

The clinical characteristics and biochemical variables of the 130 T2DM patients are presented in Table 1. Among them, 45 patients (34.6%) were diagnosed to have cfPWV of more than 10 m/s, and thus categorized as the AS group. Compared to patients in the control group, patients in the AS group were older; exhibited significantly higher SBP, DBP, body fat mass, triglyceride, fasting glucose, HbA1c, creatinine, UACR as well as FGF-21 levels; and lower eGFR.

Table 2 shows that the serum level of FGF-21 (adjusted odds ratio (aOR): 1.005, 95% confidence interval (C.I.): 1.002–1.009, *p* = 0.003), SBP (aOR: 1.004, 95% C.I.: 1.004–1.052, *p* = 0.023) and eGFR (aOR: 0.968, 95% C.I.: 0.947–0.990, *p* = 0.004) could be independent predictors for the diagnosis of AS in the T2DM patients by multivariate stepwise logistic regression analysis.

Table 3 shows that the cfPWV value was positively correlated with age (*r* = 0.238, *p* = 0.006), body fat mass (*r* = 0.195, *p* = 0.026), hypertension (*r* = 0.223, *p* = 0.008), SBP (*r* = 0.331, *p* < 0.001), DBP (*r* = 0.231, *p* = 0.008), log-TG (*r* = 0.281, *p* = 0.001), log-glucose (*r* = 0.241, *p* = 0.006), log-HbA1c (*r* = 0.218, *p* = 0.013), log-creatinine (*r* = 0.281, *p* = 0.002), log-UACR (*r* = 0.227, *p* = 0.015), and log-FGF-21 (*r* = 0.338, *p* < 0.001), but it was negatively correlated with eGFR (*r* = −0.314, *p* = 0.001) by simple linear regression analysis. Furthermore, significantly positive correlations were observed between cfPWV and SBP (β = 0.271, *p* = 0.002) as well as cfPWV and log-FGF-21 (β = 0.369, *p* < 0.001) in the T2DM patients by multivariable stepwise linear regression analysis.

**Figure 1:**
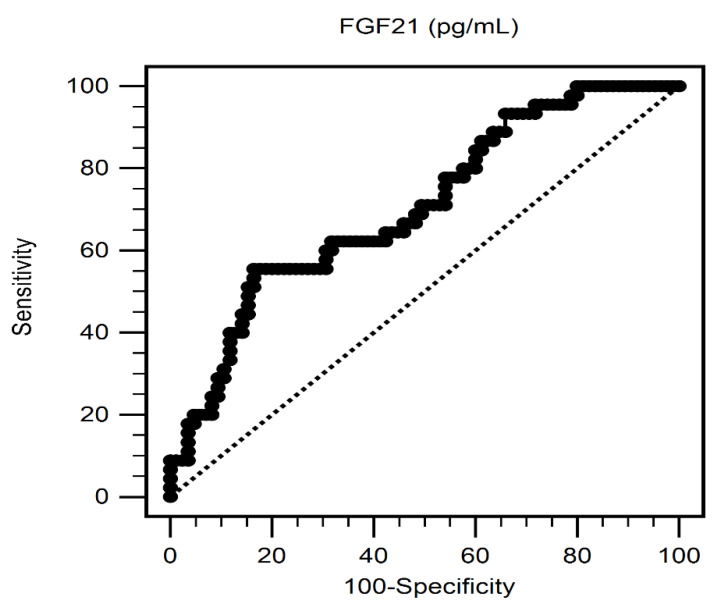
Receiver operating characteristic curve analysis of FGF-21 to predict aortic stiffness of T2DM patients. The area under the curve indicated the predictive power of FGF-21 for aortic stiffness of T2DM patients.

Figure 1 shows the optimal cut-off value of FGF-21 to predict AS of T2DM patients was 250.15 pg/mL with AUC 0.706 (95% C.I. 0.619–0.782), sensitivity 55.56% (95% C.I. 40.0–70.4%) and specificity 83.53% (95% C.I. 73.9–90.7%), respectively.

## 4. Discussion

These major findings of this study showed that higher cfPWV was positively associated with SBP and FGF-21 values and the serum FGF-21, as well as SBP and eGFR, were significant clinical predictors for the diagnosis of AS of T2DM patients.

FGF-21 is a circulating hormone produced mainly by the liver and in relatively lower amounts by other tissues, such as the pancreas and brown adipose tissue. It is a metabolic regulator with anti-inflammatory, hypoglycemic, and hypolipidemic effects according to the results of an animal study [25]. Values of seral FGF-21 have been positively correlated to several CVD-related risk factors, such as DM, obesity, metabolic syndrome, hypertension, and even brachial-ankle PWV [8,9,26]. In a longitudinal study of 87 DM patients, FGF-21 levels higher than the median level were significantly associated with an increase in CV morbidity and mortality [13]. Subsequently, another cohort study conducted in DM patients with valid FGF-21 data (9697 patients), Ong et al. showed that serum FGF-21 levels were higher in patients who were older; exhibiting higher BMI, TG levels, and creatinine levels; and had a prior history of CVD; furthermore, FGF-21 levels were associated with unsatisfactory overall CVD outcomes [12]. In another study conducted among 1996 statin-treated DM patients, those with higher baseline serum FGF-21 levels than the controls had a higher incidence of major CV events after follow-up for 1 year [11]. Longitudinal studies have shown that AS substituted by surveillance of aortic PWV could be used in the future to predict a high incidence of total CV events, CV mortality, and all-cause mortality in patients with high PWV but not in those with low PWV [27]; furthermore, the role of FGF-21 in AS has been recently studied in DM patients [10,26,28]. In cross-sectional studies on DM patients, values of seral FGF-21 were relatively high and positively correlated with high intima-media thickness of carotid arteries [26] and subclinical atherosclerosis, which is defined as an intima-media thickness of >1.0 mm or plaque of the carotid, femoral, or iliac arteries [28]. In addition, to show higher serum FGF-21 values of DM patients with subclinical atherosclerosis, Yafei et al. reported that FGF-21 was positively correlated to carotid intima-media thickness as well as cfPWV [10]. In their study, circulating FGF-21 was a significant predictor, like dyslipidemia and hypertension, of subclinical atherosclerosis [10]. In addition, FGF-21 could be used as a good biomarker for diseases such as non-alcoholic fatty liver disease, insulin resistance, dilated cardiomyopathy, and old age related cachexia [18,19,20,21]. Results of meta-analysis showed that FGF-21 serum level was remarkably higher in non-alcoholic fatty liver disease than simple steatosis as well as having a diagnostic score, odds ratio, and specificity of 1.74, 5.7, and 0.78, respectively [18]. In a cross-sectional study, serum FGF-21 level was higher in obese subjects than lean and positively corrected with insulin resistance index homeostasis model assessment [19]. Furthermore, FGF-21 level correlated negatively with left ventricular function and predict a 2.561 higher risk of mortality in a longitudinal study [20]. The serum level of FGF-21 was marked higher and correlated with cachexia in old age patients [21]. Taken together, there was a promising role of FGF-21 in coordinating the metabolic responses to reverse nutritional stress as well as a valuable biomarker for predicting a variety of metabolic disorders. In the present study, we similarly found that the values of FGF-21 were significantly positively correlated with cfPWV, and FGF-21 levels were significant predictors of AS in T2DM patients. Whether the high level of FGF-21 in patients with DM or atherosclerosis is supposed to provide protection against vascular injury or inflammation in atherosclerosis or is simply a new biomarker for CV diseases due to resistance to FGF-21 remains to be elucidated [8,29].

Studies have shown associations between AS and age as well as CV risk factors, such as DM, higher BP, hyperlipidemia, and central obesity [10,30,31,32]. Dysregulated aortic function manifests as reduced lumen diameter and premature return of the reflected late systolic wave, which can cause AS and an increase in BP, respectively [3]. Meyer et al. showed that there is a positive correlation between cfPWV and old age as well as elevated SBP [32]. Cecelja et al. found that BP and age were significantly related to cfPWV values [33]. Similarly, T2DM patients with AS in this study were older and exhibiting higher SBP and DBP than those in the control group. In addition, there is a significant positive correlation between cfPWV values and SBP even after being adjusting by covariates. Because hyperglycemia can cause structural changes in the arterial wall, cross-sectional studies have reported independent correlations in DM patients, such as between impaired glucose metabolism or high HbA1c levels and AS [30,31]. Kozakova et al. found that DM patients with suboptimal sugar control had higher BP, a higher percentage of left ventricular hypertrophy, and higher cfPWV than did patients with HbA1c levels of <6.5% [2].

Arterial stiffness induced by vascular calcification had been reported to be found in patients with impaired renal function; a meta-analysis by Sedaghat et al. showed that an increase in PWV by one standard deviation was associated with an 8% (95% CI 1.03–1.14) increase in the risk of incident CKD [34]. Regarding the association between albuminuria and renal deterioration, longitudinal studies in DM patients have shown that AS measured as cfPWV or brachial-ankle PWV was associated positively with increased severity of albuminuria [34]. Consistent with the findings of previous studies, T2DM patients with AS in this study were found to have higher body fat mass, TG levels, fasting glucose levels, HbA1c levels, creatinine levels, UACR, and lower eGFR than those with the control group. Although these factors do not persist after multivariate linear regression analysis, they were found to be significantly positively associated with cfPWV in simple linear regression analysis, which indicated that these were possible risk factors for AS in T2DM patients.

This study was limited by being conducted in a single center and enrolled only a limited number of T2DM patients. Although FGF-21 was known to be induced by nutritional inputs, including low protein diet, carbohydrates intake, fat consumption, ketogenic diets, and fasting [35], information concerning diet intake was not collected in this study. Furthermore, it was a cross-sectional study that indicated that the cause-effect relationship between serum FGF-21 levels and cfPWV could not be concluded and this requires additional longitudinal studies to confirm.

## 5. Conclusions

In this study, FGF-21 greater than was shown to be correlated with AS of T2DM patients. Valued serum FGF-21 and SBP were related to cfPWV and FGF-21 could predict AS in T2DM patients. These findings indicate that serum FGF-21 levels could be a biomarker for AS as well as play a role in the pathogenesis of AS of T2DM patients; however, the mechanism remains unknown.

## Figures and Tables

**Table 1:** Clinical variables of the 130 diabetic patients with or without aortic stiffness.

		Carotid–Femoral Pulse Wave Velocity		
Characteristics	All Patients (*n* = 130)	≤10 m/s (Control; *n* = 85)	>10 m/s (AS; *n* = 45)	*p* Value
Age (years)	62.29 ± 12.30	60.39 ± 12.35	65.89 ± 11.49	0.015 *
Male, *n* (%)	69 (53.1)	49 (57.6)	20 (44.4)	0.151
Hypertension, *n* (%)	73 (56.2)	45 (52.9)	28 (62.2)	0.310
SBP (mmHg)	142.74 ± 20.10	138.21 ± 17.62	151.29 ± 21.84	<0.001 *
DBP (mmHg)	83.15 ± 11.65	81.44 ± 11.20	86.40 ± 11.91	0.002 *
cfPWV (m/s)	9.61 ± 2.73	8.08 ± 1.40	12.51 ± 2.24	<0.001 *
Body mass index (kg/m^2^)	26.88 ± 3.95	27.72 ± 4.19	27.19 ± 3.48	0.522
Body fat mass (%)	31.71 ± 7.87	30.53 ± 8.04	33.93 ± 7.12	0.019 *
Blood urea nitrogen (mg/dL)	16.00 (12.00–19.00)	15.00 (12.00–18.00)	16.00 (12.00–22.00)	0.231
Creatinine (mg/dL)	0.8 (0.70–1.00)	0.80 (0.70–0.90)	0.90 (0.80–1.25)	0.006 *
eGFR (mL/min)	87.69 ± 26.25	94.62 ± 24.76	74.60 ± 24.12	<0.001 *
UACR (mg/g)	14.73 (6.90–52.73)	10.91 (4.95–35.04)	25.00 (9.40–151.61)	0.004 *
Fasting glucose (mg/dL)	138.00 (121.00–173.50)	130.00 (117.00–160.00)	151.00 (124.50–190.00)	0.043 *
Glycated hemoglobin (HbA1c, %)	7.50 (6.60–8.90)	7.25 (6.53–8.75)	8.10 (6.80–9.30)	0.034 *
HDL-C (mg/dL)	47.10 ± 12.23	48.00 ± 11.60	45.40 ± 13.31	0.250
LDL-C (mg/dL)	100.57 ± 26.56	101.36 ± 25.78	99.07 ± 28.21	0.641
Total cholesterol (mg/dL)	163.02 ± 29.48	163.27 ± 27.04	162.56 ± 33.95	0.896
Triglyceride (mg/dL	116.50 (85.00–171.75)	104.00 (76.50–153.50)	128.00 (84.00–212.00)	0.016 *
FGF-21 (pg/mL)	191.88 (104.57–278.52)	167.11 (95.02–233.02)	263.35 (144.21–344.05)	<0.001 *
ACE inhibitor, *n* (%)	8 (6.2)	6 (7.1)	2 (4.4)	0.555
ARB, *n* (%)	52 (40.0)	29 (34.1)	23 (51.1)	0.060
β-blocker, *n* (%)	20 (15.4)	10 (11.8)	10 (22.2)	0.116
CCB, *n* (%)	45 (34.6)	28 (32.9)	17 (37.8)	0.581
Statin, *n* (%)	68 (52.3)	42 (49.4)	26 (57.8)	0.364
Fibrate, *n* (%)	6 (4.6)	4 (4.7)	2 (4.4)	0.946
Metformin, *n* (%)	72 (55.4)	48 (56.5)	24 (53.3)	0.752
Sulfonylureas, *n* (%)	72 (55.4)	47 (55.3)	25 (55.6)	0.977
DDP-4 inhibitor, *n* (%)	78 (60.0)	51 (60.0)	27 (60.0)	1.000
Insulin, *n* (%)	35 (26.9)	23 (27.1)	12 (26.7)	0.962

Values for continuous variables are shown as means ± standard deviation tested using Student’s *t*-test; variables that did not show normal distributions are shown as medians and interquartile ranges tested using the Mann–Whitney *U* test; values are presented as number (%) and were analyzed using the chi-square test. ACE, angiotensin-converting enzyme; ARB, angiotensin receptor blocker; CCB, calcium channel blocker; cfPWV, carotid–femoral pulse wave velocity; DBP, diastolic blood pressure; DDP-4, dipeptidyl peptidase 4; eGFR, estimated glomerular filtration rate; FGF-21, fibroblast growth factor 21; HDL-C, high-density lipoprotein cholesterol; LDL-C, low-density lipoprotein cholesterol; SBP, systolic blood pressure; UACR, urine albumin-to-creatinine ratio. * *p* < 0.05 indicated statistical significance.

**Table 2:** Possible predictors of aortic stiffness of T2DM patients.

Variables	Adjusted Odds Ratio	95% C.I.	*p*-Value
Fibroblast growth factor 21, 1 pg/mL	1.005	1.002–1.009	0.002 *
Systolic blood pressure, 1 mmHg	1.004	1.004–1.052	0.023
Estimated glomerular filtration rate, 1 mL/min	0.968	0.947–0.990	0.004

Data were analyzed by multivariate stepwise logistic regression analysis (selected factors: age, body fat mass, systolic blood pressure, diastolic blood pressure, triglyceride level, fasting glucose level, glycated hemoglobin, creatinine, estimated glomerular filtration rate, urine albumin-to-creatinine ratio, and fibroblast growth factor 21). * *p* < 0.05 indicated statistical significance.

**Table 3:** Correlation between carotid–femoral pulse wave velocity levels and clinical variables among the 130 DM patients.

Variables	Carotid–Femoral Pulse Wave Velocity (m/s)				
	Simple Regression		Multivariate Regression		
	*r*	*p*-Value	Beta	Adjusted R^2^ Change	*p*-Value
Female	0.054	0.542	—	—	—
Hypertension	0.223	0.008 *	—	—	—
Age (years)	0.238	0.006 *	—	—	—
Height (cm)	−0.081	0.359	—	—	—
Body weight (kg)	0.067	0.448	—	—	—
Body mass index (kg/m^2^)	0.150	0.089	—	—	—
Body fat mass (%)	0.195	0.026 *	—	—	—
SBP (mmHg)	0.331	<0.001 *	0.271	0.065	0.002 *
DBP (mmHg)	0.231	0.008 *	—	—	—
Total cholesterol (mg/dL)	0.040	0.652	—	—	—
Log-Triglyceride (mg/dL)	0.281	0.001 *	—	—	—
HDL-C (mg/dL)	−0.082	0.355	—	—	—
LDL-C (mg/dL	−0.042	0.636	—	—	—
Log-Glucose (mg/dL)	0.241	0.006 *	—	—	—
Log-HbA1c (%)	0.218	0.013 *	—	—	—
Log-BUN (mg/dL)	0.109	0.219	—	—	—
Log-Creatinine (mg/dL)	0.263	0.002 *	—	—	—
eGFR (mL/min)	−0.314	0.001 *	—	—	—
Log-UACR (mg/g)	0.227	0.015 *	—	—	—
Log-FGF21 (pg/mL)	0.338	<0.001 *	0.369	0.168	<0.001 *

Data were analyzed by univariate linear regression analyses or multivariate stepwise linear regression analysis (the adapted factors were hypertension, age, body fat mass, SBP, DBP, log-triglyceride, log-Glucose, log-HbA1c, log-Creatinine, eGFR, log-UACR, and log-FGF21). * *p* < 0.05 indicated statistical significance.

## Data Availability

The data presented in this study are available on request from the corresponding author.
